# Persistent fifth aortic arch in a neonate with interrupted aortic arch: an unexpected intraoperative finding

**DOI:** 10.1093/icvts/ivaf145

**Published:** 2025-06-23

**Authors:** Bastien Provost, Emmanuelle Fournier, Mimi X Deng, Emre Belli

**Affiliations:** Department of Congenital Heart Diseases, Reference Center for Complex Congenital Cardiac Disease M3C, Marie Lannelongue Hospital, Plessis-Robinson, France; Division of Cardiovascular Surgery, The Labatt Family Heart Center, The Hospital of Sick Children, Toronto, ON, Canada; Department of Congenital Heart Diseases, Reference Center for Complex Congenital Cardiac Disease M3C, Marie Lannelongue Hospital, Plessis-Robinson, France; Division of Cardiology, The Labatt Family Heart Center, The Hospital of Sick Children, Toronto, ON, Canada; Division of Cardiovascular Surgery, The Labatt Family Heart Center, The Hospital of Sick Children, Toronto, ON, Canada; Department of Congenital Heart Diseases, Reference Center for Complex Congenital Cardiac Disease M3C, Marie Lannelongue Hospital, Plessis-Robinson, France

**Keywords:** aortic arch variants, interrupted aortic arch, persistent fifth aortic arch

## Abstract

Persistent fifth aortic arch (PFAA) is a rare variant of the aortic arch that may be associated with coarctation or interrupted aortic arch. We report the case of a neonate initially referred for coarctation repair. After a left thoracotomy was performed, a rare diagnosis of PFAA associated with interrupted aortic arch was made. Despite this unusual anatomy, the repair was successfully performed via a lateral approach. This unusual anatomy of the aortic arch deserves special consideration in case of association with coarctation. Indeed, repair from the side may not be possible due to the common origin of the neck-vessels, and resection should be extended as far as possible to eliminate remaining ductal tissue and prevent recoarctation.

## INTRODUCTION

First described by Van Praagh as a double-lumen aortic arch in 1969, persistent fifth aortic arch (PFAA) is a rare and difficult to diagnose congenital malformation of the aortic arch [1]. Weinberg later classified PFAA into three types:

Type A: associated with a patent original aortic arch,

Type B: associated with an atretic or interrupted arch, and

Type C: forms a systemic-to-pulmonary connection, mimicking an aortopulmonary window [2].

Histologically, the PFAA tissue in cases of associated coarctation resembles ductal tissue with disorganized elastic fibres and smooth muscle cells [3]. Thus, identifying the presence of a PFAA is mandatory to resect the abnormal segment and to further prevent recurrence of coarctation.

## CASE PRESENTATION

A 5-day-old neonate was referred for repair of a presumed isolated coarctation, based on clinical findings of a systolic murmur and a 25 mmHg pressure gradient between the upper and lower extremities. Transthoracic echocardiography revealed a narrowed aortic segment (1.3 mm) with a posterior shelf and typical flow acceleration (Video 1). Notably, there was an unusual common origin of the head-neck vessels.

**Figure 1:**
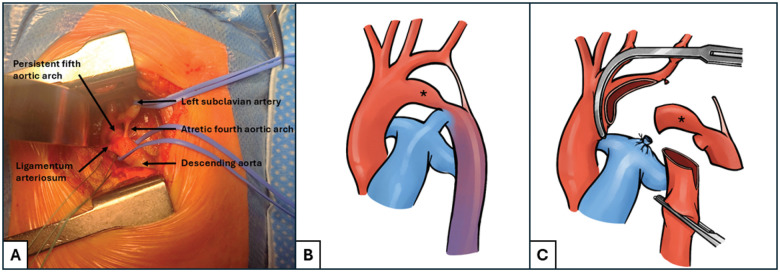
(**A**) Intra-operative view of the persistent fifth aortic arch through left thoracotomy, associated with an interrupted aortic arch. (**B**) Illustration showing the common origin of the neck-vessels, the atretic fourth aortic arch, and the persistent fifth aortic arch (*). (**C**) Cross-clamps were applied both ends, the persistent fifth aortic arch/coarctation site was resected and an extended end-to-end anastomosis was performed.

After performing a left thoracotomy for coarctation repair, the diagnosis was confirmed. In addition to the coarctation, an atypical fibrotic segment was identified between the left subclavian artery and the descending aorta, corresponding to an atretic fourth aortic arch and thus consistent with an interrupted aortic arch (Fig. 1A). What initially appeared to be a normal aortic arch was eventually identified as a persistent fifth aortic arch connecting the ascending and the descending aorta, with associated coarctation. This anatomical configuration corresponded to a Type B PFAA according to Weinberg’s classification (Fig. 1B).

After cross-clamping both ends, first the PFAA/coarctation was extensively excised, then the incision was enlarged to perform a wide extended end-to-end anastomosis (Fig. 1C).

Discharge echocardiogram showed no residual gradient across the aortic arch. A follow-up echocardiogram after 6 months revealed again no residual gradient with the patient being asymptomatic.

## DISCUSSION

PFAA is a rare but surgically relevant vascular variant, which needs to be identified ideally prior to surgical treatment. In this case, the preoperative identification of a common origin of all head and neck vessels was highly suggestive of a Type A interrupted aortic arch. However, the presence of a persistent connection between the ascending and descending aorta argued against this diagnosis.

The intraoperative identification of a fibrotic connection between the left subclavian artery and the descending aorta confirmed the diagnosis of an associated interrupted aortic arch, so that the segment connecting the ascending and descending aorta could only be a persistent fifth aortic arch (PFAA). This dual arch system, where the fourth aortic arch is interrupted and the fifth forms the functional aortic arch, poses both diagnostic and technical challenges. Cross-sectional imaging such as computed tomography or cardiac magnetic resonance imaging might have clarified the anatomy preoperatively. In our case, the typical clinical presentation of neonatal coarctation deterred us from performing further exams, which would have been helpful to anticipate the repair.

Histologically, coarctation-associated PFAA often exhibits ductal-like tissue, underscoring the importance of complete excision to prevent restenosis. The absence of a residual gradient at 6-month follow-up supports the durability of our strategy.

This case highlights the need for heightened awareness of PFAA among congenital cardiac surgeons. Atypical arch anatomy, especially with a common origin of supra-aortic vessels, warrants detailed imaging and careful intra-operative exploration. Recognizing and appropriately managing PFAA is essential for optimizing long-term outcomes in these complex neonatal patients.

## Data Availability

The data underlying this article are available in the article.

## ABBREVIATIONS

PFAA: Persistent fifth aortic arch
